# Prediction of biochemical failure in localized carcinoma of prostate after radical prostatectomy by neuro-fuzzy

**DOI:** 10.4103/0970-1591.30258

**Published:** 2007

**Authors:** Neeraj Kumar Goyal, Abhay Kumar, Rajiba L. Acharya, Udai Shankar Dwivedi, Sameer Trivedi, Pratap Bahadur Singh, T. N. Singh

**Affiliations:** Department of Urology, Institute of Medical Sciences, Banaras Hindu University, Varanasi - 221 005, India; * Department of Earth Sciences, Indian Institute of Technology, Mumbai - 4000 76, India

**Keywords:** Neuro-fuzzy, prostate cancer, radical prostatectomy

## Abstract

**Objective:**

To predict biochemical failure in localized prostate cancer after radical prostatectomy using preoperative variables.

**Materials and Methods:**

Twenty-six patients of early carcinoma of prostate underwent open retropubic radical prostatectomy from June 2002 to June 2006. Preoperative variables included age, family history, digital rectal examination, serum prostatic specific antigen (S. PSA), prostate biopsy Gleason score, MRI of pelvis variables like periprostatic extension, seminal vesical invasion, weight of gland and pathological stage. With application of neuro-fuzzy, these variables were fed into system as input and output, that is S. PSA at six months (predicted value) was calculated. Neuro-fuzzy system is a system to combine fuzzy system with learning techniques derived from neural networks. Here, we applied Takagi Sugeno Kang model (TSK) due to its close solution to our aim. All the patients were followed up for a minimum of six months. At six month S. PSA of all patients was done (observed value). Predicted and observed values were compared.

**Result:**

Predicted and observed values were plotted on 1:1 slop line. Coefficient of correlation was 0.9935.

**Conclusion:**

Coefficient of correlation is close to one. It indicates that the neuro-fuzzy is accurate in predicting biochemical failure in localized carcinoma of prostate after radical prostatectomy.

For organ-confined disease in carcinoma of prostate, various treatment options are available including watchful waiting, radiation therapy or retropubic radical prostatectomy. Out of these retropubic radical prostatectomy has been established as the primary curative procedure.[1] It is the most frequently performed procedure used in 52% of patients, followed by external radiotherapy or brachytherapy, which is used in approximately 20% of patients. Despite definitive therapy approximately 40% of the patients demonstrate disease recurrence.[2] Most of the relapses occur in the first five years.[3] Thus it is important to have a reliable method to predict cancer progression after radical prostatectomy to decide rationally whether individual patients should be treated with an adjuvant therapy scheme. In general, the prediction of prostate cancer relapse may be based on routinely available data only (e.g., tumor grade, clinical stage and preoperative S. PSA levels). We used neuro-fuzzy as a prediction tool to predict prostate cancer progression after radical prostatectomy.

## MATERIALS AND METHODS

**Figure 1:**
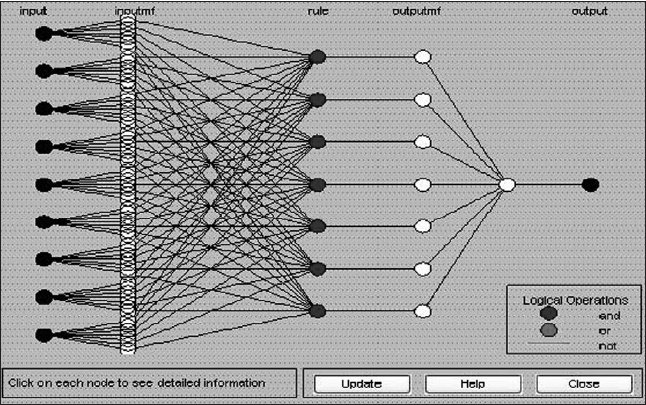
Neurofuzzy neural network

**Figure 2:**
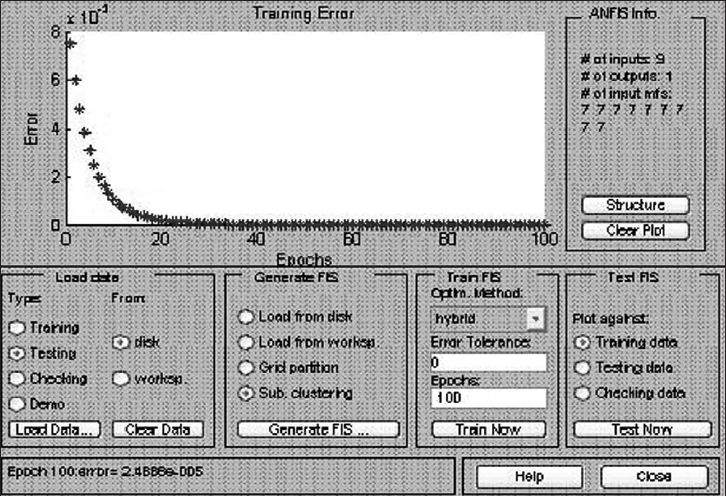
Neurofuzzy under training and error estimation

From June 2002 to June 2006, 26 patients of localized carcinoma prostate have undergone radical retropubic prostatectomy in our department. Seven cases were lost to follow-up and were excluded from the study. All patients were evaluated thoroughly by history and physical examination, especially digital rectal examination (DRE). Routine investigations included hemogram, blood biochemistry (blood urea, blood sugar, serum creatinine, serum electrolytes), liver function test, urine routine examination and culture-sensitivity, X-ray chest and ECG along with serum prostatic antigen (S. PSA), Prostate Biopsy and Gleason's grade and score, MRI of pelvis (variables included periprostatic extension, seminal vesical invasion and pelvic lymph nodes status). Open retropubic radical prostatectomy was done in all patients. All patients were followed up at three-monthly with S. PSA, level minimum for six months. We trained the neuro-fuzzy system with the first 16 cases [Table 1]. In this the input variables and output data was known and was fed into the system to train it [Figure 1]. Input data included age, family history, digital rectal examination, S. PSA, prostate biopsy Gleason score, MRI of pelvis variables like periprostatic extension, seminal vesical invasion, weight of gland and pathological stage. Then two or three cases from the training set were used for validation (validation set). Once validation was found satisfactory further training was stopped to prevent “over training” of the network system [Figure 2]. Once neuro-fuzzy was fully trained, input data from subsequent four patients were fed and S. PSA, at six months (predicted values) was calculated. These patients were followed up for a minimum of six months after radical prostatectomy to know the actual S. PSA, value at six months (observed values). Then observed and predicted values were compared to draw the inference.

**Table 1:** Patients' details

Age	FH	S. PSA	BxGS	DRE	MRI-weight	MRI-ECE	MRI-SV	Path stage	S. PSA 6 m
68	-	6.7	9	N	50	-	-	2	0
56	-	10	7	N	36	-	-	2	0.1
59	-	6.6	4	N	40	-	-	2	0.12
73	-	7.8	8	N	45	-	-	2	0.15
65	-	9.9	7	N	30	-	-	2	0.2
60	-	27	4	Abn	37	-	-	3	0.24
71	-	9.4	8	N	35	-	-	2	0.32
65	-	13	6	N	38	-	-	2	0.4
64	-	13	7	Abn	37	+	-	2	0.52
52	-	8.9	8	N	46	-	-	2	0.56
62	-	5.7	8	N	33	-	-	3	0.85
50	-	7.8	6	N	90	-	-	4	2.46
66	-	10	4	N	25	-	-	2	0.24
67	-	11	8	N	30	-	-	2	0.4
60	-	17	4	N	35	-	-	2	0.11
50	+	15	4	N	32	-	-	2	0.2
55	-	11	8	N	30	-	-	3	0.62
50	-	7.8	5	N	32	-	-	2	0.25
51	-	6.5	4	N	30	-	-	2	0.2

FH - Family history, S. PSA - Serum prostate specific antigen, BxGS - Biopsy gleason score, DRE - Digital rectal examination, MRI - weight- Magnetic resonance imaging of pelvis- weight of prostate, MRI-ECE- Magnetic resonance imaging of pelvis - Extracapsular extension, MRI-SV- Magnetic resonance imaging of pelvis - Seminal vesical invasion, Path stage - Pathological stage, N- Normal, Abn- Abnormal, - = Not present, += Present

## RESULT

**Figure 3:**
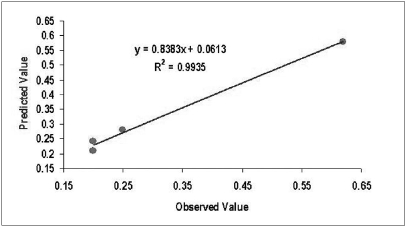
Results plotted on 1:1 slope line & co-efficient of correlation

Predicted and observed values of S. PSA at six months after radical prostatectomy are shown in Table 2. We calculated the coefficient of correlation (COC) (r^2^) with the available data. The COC was 0.9935. Predicted and observed values were also plotted on 1:1 slop line as shown in Figure 3.

**Table 2:** Predicted and observed values of S. PSA at six months

Case No.	Predicted value of S. PSA	Observed value of S. PSA
1.	0.24	0.2
2.	0.58	0.62
3.	0.28	0.25
4.	0.21	0.20

S. PSA - Serum prostatic specific antigen

## DISCUSSION

Most of the literature on localized carcinoma of prostate is from the western world. There are no standard Indian guidelines regarding management of localized carcinoma of prostate. We are performing open retropubic radical prostatectomy in our center as the first treatment for localized prostate cancer. However, all the available treatments may exert a significant impact on the patient's health-related quality of life. Therefore one must identify those patients who might do well with active surveillance, who should receive multimodal therapy and who should be treated presumptively for advanced disease. Numerous algorithms and nomograms have been developed which predict pathological stage and disease progression (biochemical failure). Ross *et al* in 2001[4] counted 42 nomograms for risk assessment at various stages of prostate cancer. After that many more have been published. In general the prediction of tumor grade, clinical stage and preoperative PSA levels or these data may be combined with data from additional techniques like flow cytometry, image cytometry, morphometry stercology and molecular biology.

We use neuro-fuzzy to predict biochemical failure after radical prostatectomy. Fuzzy set theory exhibits immense probabilities for the effective solving of the uncertainty in the problems. Fuzziness means ‘vagueness’. Fuzzy set theory is an excellent mathematical tool to handle the uncertainty arising due to vagueness. It is a widely used tool in various fields of science and engineering. Since the design and especially the optimization process of fuzzy systems can be very time-consuming, it is convenient to algorithms, which construct and optimize them automatically. One popular approach is to combine fuzzy system with learning techniques derived from neural networks. Such approaches are called neuro-fuzzy system. The most popular solution of the fuzzy networks is based on the so-called inference system, fuzzy ‘if – then’ rules and fuzzy reasoning. Such a fuzzy inference system implements a nonlinear mapping from input space to output space. This mapping is accomplished by a number of fuzzy ‘if – then’ rules, each of which describes the local behavior of the mapping, like it is done in radial basis function networks. The antecedent of the rule defines the fuzzy region in the output space, while the consequent specifies the output of the fuzzy region. There are different solutions of fuzzy inference system. Two well-known fuzzy modeling methods are the tsukamoto fuzzy model and takagi sugeno kang model (TSK). Here, we applied TSK model due to its close solution to our aim.

A lot of work has already been done to predict disease progression by statistical tools[5,6] as well as by artificial neural network.[7,8] Recently, Luigi Benecchi[9] used the neuro-fuzzy system to diagnose prostate cancer and compared its predictive accuracy with that obtained by total PSA and percent-free PSA. He concluded that the predictive accuracy of the neuro-fuzzy was superior to that of the PSA and %f PSA. James W.F compared the neuro-fuzzy model and artificial neural network (ANN).[10] He concluded that neuro-fuzzy had a similar or superior predictive accuracy to ANN. Although we did not draw the results with other statistical methods, the coefficient of correlation was very close to 1, indicating its very good predictive accuracy.

This is a pilot study. The pitfalls of the study are: the number of cases is less, follow-up period of patients is short. We presented the preliminary results of the study.

## CONCLUSION

For prediction, more close the value of coefficient of correlation to 1, more accurate the system is. In our case r^2^ is 0.9935 that is very close to 1. It indicates that the system has very good prediction capability. The preliminary results of our study are encouraging. More number of patients and comparative analysis with other statistical tools will be done in future to establish it as the most accurate tool for prediction.
